# Construct Validity and Dimensionality of the Rosenberg Self-Esteem Scale and Its Association with Spiritual Values Within Irish Population

**DOI:** 10.1007/s10943-019-00821-x

**Published:** 2019-05-03

**Authors:** Krzysztof Kielkiewicz, Ciarán Ó. Mathúna, Christopher McLaughlin

**Affiliations:** 1 Department of Psychology, University of Finance and Management, Warsaw, Poland; 2 PCI College, Dublin, Ireland; 3 Department of Education, Marino Institute of Education, Dublin, Ireland; 4 Department of Business, Sligo Institute of Technology, Sligo, Ireland

**Keywords:** Self-esteem, Spirituality, SEM, Ireland

## Abstract

Until this research, correlation of Rosenberg’s (1965) self-esteem scale (RSES), with religious and spiritual values, was never investigated using the measure as a twofold construct instead of the monolithic form. This research paper explores the prediction of RSES by spiritual values using a twofold structure of: self-esteem-positive (SEP) and self-esteem-negative (SEN), to specify individual and fragmented correlations with spirituality, which until now was unobtainable. Confirmatory factor analysis and structural equation modelling were applied to analyse the data. The survey was conducted among two-hundred and sixty-eight participants from the Republic of Ireland. The research finds that spirituality influences peoples’ self-esteem, but clear and linear correlation between spirituality and self-esteem is difficult to be concluded. People respond oppositely and differently to positive and to negative items in the RSES which indicates that the measure is a combination of two statistically consistent constructs: SEP and SEN. The results of the study confirm that there are many spiritual areas that affect self-esteem.

## Introduction

Rosenberg’s self-esteem scale (RSES) was developed as a measure of subjective self-evaluation in 1965 (Rosenberg 1965) as a ten-question Likert-type scale. This construct is very often used in social survey questionnaires, especially within fields of sociology and psychology. The scale comprises of five positive and five negative statements. Recent debate is centred on its use as an all-inclusive construct or as a twofold variable scheme. The studies of Debowska et al. (2017), Sharratt et al. (2014), Boduszek et al. (2013), and Boduszek et al. (2012) suggest that the twofold structure is a more appropriate manner of applying RSES in social studies. Recent research applying the bifactorial modelling also confirmed the two-dimensional nature of the self-esteem construct (Hyland et al. 2014; McKay et al. 2014). Their argument is supported by previous research of Kaufman and Rasinski (1991), Goldsmith (1986), Bachman and O’Malley (1986), Carmines and Zeller (1979), Dobson et al. (1979), Hensley (1977), Kaplan and Pokorny (1969). The study of Kaufman and Rasinski (1991) suggests that respondents react differently to the positive and negative statements of RSES. According to Hyland et al. (2014), people have different associations with positive and negative expressions. Debowska et al. (2017) conclude that high SE associates with different psychological spans than its relative low SE.

RSES is regularly used to facilitate investigation in the areas of religion (Krause 2009; Rothschild et al. 2009; George et al. 2002; Krause 1995) and spirituality (Abdel-Khalek 2011; Bradley et al. 2005; Emmons et al. 1998). Often both of these terms, religion and spirituality, are used as a one structural construct or even interchangeably (Reiland and Lauterbach 2008; Francis and Kaldor 2002; Krause 2003; Laurencelle et al. 2002; Krause 1995; Ellison 1993). Spirituality, as a discipline under development, is today on a path to its self-identification and recently appears to be defined in various ways through its association with diverse religious, social, and professional domains. The existing literature illustrates its difficulty in having a commonly accepted characterisation (Greenstreet 1999; Hicks 2003). Recent definitions often appear to be disharmonic or even conflicting (Johnson et al. 2004; Lemmer 2002; Tisdell 2001; Marcic 2000; Laurence 1999; Narayanasamy 1999). Some authors suggest that the reason for this is that the subject matter of spirituality is deeply subjective and that the concept is very diffuse (Harlos 2000). However, it is now accepted that spirituality is an academic discipline (Frohlich 2001) and its development is also perceived as area that overlaps and intertwines with theology and psychology (Helminiak 1996). For the purpose of this paper, the authors have consciously decided not to provide a definition of spirituality as one has not been agreed upon within academia. The authors are also conscious that there are a large number of definitions dependent on the domain in which they function.

The current literature connects spirituality with various academic and professional domains; among them are mental health (Hill and Pargament 2008; Plante and Sherman 2001; Larson et al. 1998) and well-being. The research shows that well-being interacts with the domain of spirituality, and it could be understood as a subjective evaluation of someone’s life (de Souza 2009; Meraviglia 2004; Cohen 2002; Daaleman et al. 2001; Diener 2000). Previous studies of spirituality found its fundamental association with God, prayer, and faith (Sheldrake 2009; Hyman and Handal 2006; Walton 1999; Reed 1987; Bahr and Martin 1983; Benson and Spilka 1973). These represent a transcendent dimension of the phenomenon. Nevertheless, spirituality relates to practice within the secular dimension in a form of communal life (Huls and Waaijman 2005; Johnson 2001), along with a relationship to the material world and Material Values (Miller and Martin 1988). Taves (2003) underlined meaning of formation as a crucial aspect of spirituality. Young, Cashwell and Woolington (1998) explored the relationship of spirituality to cognitive and moral development. Zinnbauer et al. (1997) were pioneers of quantitative approach and investigated the ‘*fuzzy’* concept of spirituality (Zinnbauer et al. 1997), which is also characterised by the sacrifice of one’s own self, for the needs of others.

Zaleski (2002) and Hayles (2002) in their studies disclose how current technology influences peoples’ perception of spirituality and religiosity. Multimedia accelerates communication globalisation, information, and cultural exchange. Hayles (1993) investigated this aspect previously from the aspect of how virtual reality influences a person’s spiritual life. She conceptualised the presence of a virtual world within the field of spiritual investigation as new phenomenon. Maas (2005), when investigating current spirituality within a context of postmodern philosophy, paid attention to how self-esteem associates with the search for meaning in life. Similarly, Conway (2007) underlines the importance of a sense of life, in relation to spiritual development. Many scholars suggest that dialogue with self, along with deed, is very meaningful in the context of current spirituality (Banathy and Jenlink 2005; Pesut 2003; Spohn 2001; Liebert 2002), as these are understood as an embodiment of spiritual growth. The findings of research by Levin and Tobin (1995), Zika and Chamberlain (1992), Poloma and Pendleton (1990, 1991) suggest that engagement in religious and spiritual life has a positive influence on psychological well-being.

The investigation of correlation of these two areas seems to be reasonable and appealing, especially in light of the fact that in spite of extended previous studies of relationship between SE and spirituality, the application of SE as a twofold construct was never applied. Analysing previous findings, there is a strong premise that people associate different spiritual values with SEP and SEN. Therefore, the current exploration is not just interesting, but a required complement to the research in spirituality, in the light of recent findings on SE as twofold construct, hence the purpose of this paper. In accordance with recent findings (Debowska et al. 2017; Hyland et al. 2014; McKay et al. 2014; Boduszek et al. 2012, 2013), RSES will be applied as a twofold structure divided into self-esteem positive (SEP) and self-esteem-negative (SEN). All the above domains associated with spirituality will be implemented into the investigation of their prediction on self-esteem.

## Method

### Participants

The sample included 268 participants recruited in the Republic of Ireland. The opportunistic sample consisted of 175 females (65.3%) and 91 males (34%). Two participants did not indicate their gender. The respondents ranged in age from 18 to 78. The average age of participants was 32.65 (SD = 12.97). Most of the participants (72.4%; *n* = 194) came from urban areas of Ireland. 4.5% (*n* = 12) of participants reported having primary school education, 8.6% (*n* = 23) had secondary school education, 23.5% (*n* = 63) had not completed college or university, and 63.1% (*n* = 169) had completed college/university education. 2.2% (*n* = 6) did not provided information about their education. 62.3% (*n* = 167) of the participants indicated their marital status as single, 24.6% (*n* = 66) as married, 6% (*n* = 16) as divorced or separated, and 1.1% (*n* = 3) as widower. 6% (*n* = 16) did not provided information about their marital status. The group of participants comprised 53.7% of Roman Catholics (*n* = 144), 8.6% of Protestants (*n* = 23), 6.3% other Christians (*n* = 17), 10.4% of participants classified themselves as a believer but not religious (*n* = 28), and 5.6% as Atheists (*n* = 15). 4.9% did not provide information about their religious classification (*n* = 13). 53.4% (*n* = 143) rated themselves as spiritual, 9.3 (*n* = 25) as non-spiritual, and 31.3% (*n* = 84) as neither spiritual nor non-spiritual. 6% (*n* = 16) did not provided information on spiritual classification.

### Measures

All of the responses, dependent and independent variables, were measured on 4-point Likert scale (SA—strongly agree, A—agree, D—disagree, and SD—strongly disagree).

### Dependent Variables

The original *self-esteem* scale (Rosenberg 1989) comprised of ten Likert-type scale items. For this study, the twofold construct was used. Five of them constructs were from the *self-esteem positive* (SEP) (Cronbach’s *α* = .66), and the other five were from the *self-esteem-negative* (SEN) (Cronbach’s *α* = .79). The *self-esteem* scale explores positive and negative evaluations of participants towards one’s own self.

1. *Self-esteem positive* (SEP) factor is measured by five questions:

1. I take positive attitude towards myself
2. I am able to do things as well as most other people
3. On the whole, I am satisfied with myself
4. I feel that I have a number of good qualities
5. I feel that I am a person of worth, at least on an equal plane with others

2. *Self-esteem-negative* (SEN)

1. I certainly feel useless at time
2. At times, I feel I am not good at all
3. Generally, I am inclined to feel that I am a failure
4. I wish I could have more respect for myself
5. I feel I do not have much to be proud of

### Independent Variables

1. The factor *God* refers to the existence of personal or philosophical absolute being within a transcendent reality. *God* refers to the infinite being and was measured by three questions (Cronbach’s *α* = .76):

1. God is someone who loves me the most
2. God is an idea which does not exist in reality
3. It is possible to live according to God’s will

2. *Prayer* communicates the level of a participant’s engagement in private prayer. This factor was examined by two questions (Cronbach’s *α* = .91):

1. A prayer is a part of my everyday practice
2. Prayer helps me to deal with my personal problems

3. *Spiritual Formation* represents people’s conviction that spirituality is an important component of human formation. Spirituality should be intrinsic to the development of human character and personal growth. Formation was measured by (Cronbach’s *α* = .79):

1. Spiritual life should head towards practical personal improvement
2. Spiritual life should develop personality
3. Spirituality is also about formation of human’s character

4. The factor *Spiritual Being* refers to human understanding and ability to sacrifice in one’s own life as being a valuable and desired quality of life. The goal of spiritual development in light of *Spiritual Being* is an ability to dedicate of own life for others. The questions used to examine this were (Cronbach’s *α* = .82):

1. Authentically spiritual person does a lot for others
2. Spirituality helps to distance of own selfishness and egocentrism to be more helpful for others
3. Spiritual people can do more for others than non-spiritual persons

5. *Faith* is concerned with the human relationship with God. This represents the divine religious or/and spiritual reality, which is unreachable physically. *Faith* refers to belief in a reality that functions beyond time and mater, without proof or evidence. Faith is very often associated with religious doctrines, however, not necessarily. *Faith* was measured by three questions (Cronbach’s *α* = .89):

1. Faith is important in my life
2. Most of the time, the faith helps me to cope with my everyday problems
3. Faith helps me to evaluate my life

6. *Online Being* reflects people’s socialising and time spent online. This factor was measured by questions (Cronbach’s *α* = .66):

1. I participate in online chatting
2. I have many friends; however, I meet most of them only online
3. I like to spend my free time online

7. The *Community* factor reflects attitudes regarding social interactions. This factor was measured by three questions (Cronbach’s *α* = .66):

1. Life is most worthwhile when is lived in service to other people
2. We need each other to stay psychically healthy
3. Community is an important part of every person’s normal life

8. *Material Values* is the factor which reflects material things that exist in the world and are used by people in everyday life. This factor was measured by two questions (Cronbach’s *α* = .71):

1. Money is very important to me
2. Money is something I cannot imagine my life without

9. The factor *Sense of Life* refers to the conviction that human life makes sense when it is dedicated to the search for the meaning of life. This factor represents a theoretical assumption in which people believe, but also personal association with this assumption as a life value. *Sense of Life* was measured by (Cronbach’s *α* = .85):

1. Finding of the meaning and purpose of life is one of the most important goals in our life
2. Life is only worthwhile when is a search for the sense of life
3. Life without a search for the meaning and purpose is not much worth

10. *Happiness* represents belief that Happiness, contentment, or pleasure is the ultimate purpose of human life and they are the strongest drives of people’s attitudes and decisions. Happiness was measured by three questions (Cronbach’s *α* = .62):

1. I believe that finding Happiness in life is more important than finding the sense of life
2. Do you agree that heading towards Happiness is the most important in life?
3. Everyone just wants to be happy, even if others need to suffer because of it a little

11. *Dialog with Self* reflects appreciation of self-reflection and dialog with one’s own self as an important part of spiritual development. This factor was measured by four questions (Cronbach’s *α* = .70):

1. Being yourself is more valuable than being rich
2. Dialogue with one’s own self is an important aspect of spiritual life
3. Spiritual life cannot exist without honest dialogue with own self
4. Without inner dialogue with self, it is difficult to evaluate own life

12. *Spiritual Deed* is the simple ability of undertaking constructive actions or activities stimulated by spiritual motives. *Spiritual Deed* was measured by three questions (Cronbach’s *α* = .73):

1. An authentic spiritual life can be verified only by good deed
2. An immoral life disproves an authentic spiritual life
3. An authentic spiritual life always results in moral success

### Procedure

The data were collected opportunistically among participants living in the Republic of Ireland at the time of the study. Participation was voluntary, and respondents were recruited from among students of: Dublin Business School, visitors of Offaly Public Libraries (Tullamore, Birr, Clara, Ferbane, Kilcormac, Banagher), Tullamore Parish of the Church of Ireland, students and staff of ICPPD College in Athlone, Adult Education Centre in Athlone, and from teachers in the Primary School in Kilbeggan. The survey questionnaire was also available online. Participants of the survey were recruited according to prior agreement of the authorities of the above institutions and were informed about the voluntary nature of the study. The participants were also informed about the anonymity of their responses, and the questionnaires were returned in sealed envelopes.

### Analysis

Preliminary analysis was conducted through SPSS 21 to ensure that the data were suitable for structural equation modelling. Additionally, descriptive statistics and the Pearson product–moment correlation coefficient were analysed between all the continuous variables. Further analysis contained two levels: measurement and structural. In relation to the measurement level, two different models of self-esteem construct were specified and estimated in Amos 21 using confirmatory factor analysis (CFA) technique. This helped to determine the factor structure, and factor loadings of measured variables, and to assess the fit between the data and pre-established theoretical models. A covariance matrix was computed, and the parameters were established using a maximum likelihood. Goodness-of-fit indices were used to assess the fit of the model: Chi-square (*χ*^2^), Root Mean Square Error of Approximation (RMSEA; Steiger 1990) with 90% confidence interval (90% CI), Comparative Fit Index (CFI; Bentler 1990), and Incremental Fit Index (IFI; Bollen 1989). A nonsignificant Chi-square (Kline 2005) and values above .95 for the CFI and IFI are considered to reflect a good model fit (Vandenberg and Lance 2000; Hu and Bentler 1999). However, for CFI and IFI, values above .90 indicate adequate fit (Hu and Bentler 1999; Bentler 1990). RMSEA values less than .05 suggest good fit, and values up to .08 indicate reasonable errors of approximation in the population (Browne and Cudeck 1993).

**Fig. 1:**
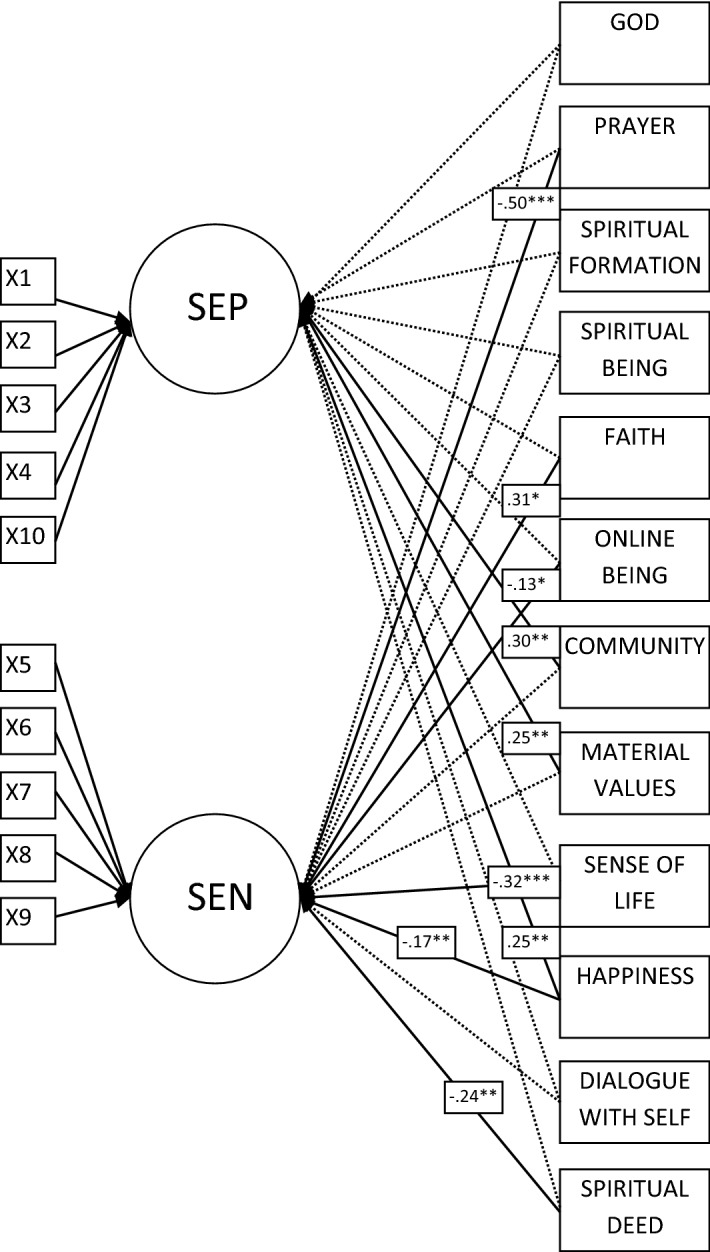
Model of association of self-esteem with spiritual values (ASESV). X question; SEP self-esteem positive; SEN self-esteem negative

In terms of the structural level, the conceptual model of the *Model of Association of Self*-*Esteem with Spiritual Values* (Fig. 1) was specified and estimated in Amos 21 with a maximum likelihood estimation using structural equation modelling (SEM). SEM is a broad data analytic method for the quantification and statistical testing of theoretical constructs. The common structural equation model is a combination of two data analytic methods: path analysis (PA) and factor analysis (FA). PA is a technique of pictorially demonstrating the associations among observed variables in a path diagram. Thus, within a SEM method, the structural and measurement elements of analysis are estimated simultaneously (MacCallum and Austin 2000). In the current research, the structural part of the analysis determines the relationship between two latent variables (self-esteem positive and negative) and observed predictors.

## Results

### Descriptive Statistics and Correlations

Descriptive statistics including means (M) and standard deviations (SD) for the all variables are presented in Table 1, together with Cronbach’s alpha reliability (Cronbach 1951).

**Table 1:** Descriptive statistics for all continuous variables included in the study

Scale	M	SD	Cronbach’s alpha (*α*)
God	8.27	2.44	.76
Prayer	5.09	2.06	.91
Spiritual Formation	8.12	2.06	.79
Spiritual Being	7.45	2.23	.82
Faith	8.24	2.70	.89
Online Being	6.62	2.09	.66
Community	9.38	1.61	.66
Material Values	8.71	1.52	.71
Sense of Life	7.76	2.33	.85
Happiness	8.37	1.64	.62
Dialog with Self	11.86	2.18	.70
Spiritual Deed	7.49	2.08	.73
Self-Esteem (SE)			
SE Positive	16.24	1.95	.66
SE Negative	14.28	3.20	.79

The relationships between all variables were investigated using Pearson’s product–moment correlation coefficient in order to test if the variables are sufficiently correlated to be included in one structural model (see Table 2). Additionally, preliminary analyses were performed to ensure no violation of the assumptions of normality, linearity, and homoscedasticity.

**Table 2:** Correlations between all continuous variables

Variables	SEN	SEP	G	F	COM	MV	UT	HAP	SAC	FOR	PRA	DS	D	B
SEN	–													
SEP	.28***	–												
God (G)	− .13*	18**	–											
Faith (F)	− .20*	.16**	.80***	–										
Community (COM)	− .05	.32***	.37***	.40***	–									
Material Values (MV)	− .07	.24***	.08	.15*	.41***	–								
Sense of Life (SOL)	− .23***	.12	.49***	.54***	.38***	.12	–							
Happiness (HAP)	− .21***	.22***	− .03	.10	.14*	.26***	.14*	–						
Spiritual Being (SB)	− .23***	.12	.61***	.61***	.41***	.07	.70***	.09	–					
Spiritual Formation (SF)	− .19**	.18**	.53***	.57***	.42***	.12	.64***	.13	.71***	–				
Prayer (PRA)	− .25***	.09	.78***	.83***	.34***	.06	.50***	.50***	.58***	.57***	–			
Dialog with Self (DS)	− .23***	.19**	.49***	.50***	.36***	.04	.55***	.15*	.60***	.65***	.46***	–		
Spiritual Deed (SD)	− .26***	.07**	.60***	.67***	.39***	.18**	.64***	.10	.70***	.71***	.61***	.57***	–	
Online Being (OB)	− .14**	− .03**	− .96	− .06	− .03	.15*	.08	.14	.04	.00	− .06	.02	.07	–

Significance level: *p < .05; **p < .01; ***p < .001

### Construct Validity and Dimensionality of Self-Esteem Measure

Construct validity and dimensionality of self-esteem were vitally important to test, particularly, regarding the appropriate incorporation of latent construct in the structural equation model. The analysis involved comparing the two alternative confirmatory factor analyses (CFA) of self-esteem. The two models include: a one-factor model (all items) and a two-factor model (self-esteem positive comprising of items SelfEs1, SelfEs2, SelfEs3, SelfEs4, SelfEs10, and self-esteem negative comprising of items SelfEs5, SelfEs6, SelfEs7, SelfEs8, SelfEs9). The specified models in this study allowed items to load only onto a single factor, with uncorrelated measurement error terms as suggested in previous research (Debowska et al. 2017; Hyland et al. 2014; McKay et al. 2014; Boduszek et al. 2012, 2013). Table 3 presents both absolute and comparative fit indices for each model. Furthermore, Akaike Information Criterion (AIC) (Akaike 1974) was used to evaluate two alternative models, with the smaller value demonstrating the best fitting model. Findings suggest that the two-factor model provided a better fit for the self-esteem items than the one-factor model. As shown in Table 3, all indices show improvement in the two-factor model. Although the *Chi-square* statistic was significant, the CFI, TLI, and RMSEA all indicated satisfactory fit. The AIC also shows that the two-factor model is a better model compared to the one-factor model. Table 4 reports the standardised and unstandardised factor loadings for each item on their respective factor. All of the item loadings were between .30 and .79 on the self-esteem positive factor and between .58 and .71 on self-esteem negative factor.

**Table 3:** Fit indices for the alternative CFA models of spiritual values and Rosenberg’s self-esteem scale

Item	1-Factor model	2-Factor model
*χ*^2^	258.57	78.05
*df*	35	34
*p*	.00	.00
RMSEA	.16	.07
90% CI	.14 .17	.05 .09
AIC	318.57	140.05
CFI	.63	.93
IFI	.64	.93

*RMSEA* Root Mean Square Error of Approximation, *CI* confidence interval, *SRMR* Standardised Root Mean Square Residual, *AIC* Akaike Information Criterion, *CFI* Comparative Fit Index, *IFI* Incremental Fit Index

**Table 4:** Standardised and unstandardised regression weights (with standard errors) for the specified structural equation model of prediction spiritual values on self-esteem

Variables	*B*	S.E.	*β*
Structural level			
SEP ← God	.009	.011	.097
SEN ← God	.022	.027	.088
SEP ← Prayer	− .009	.013	− .084
SEN ← Prayer	− .145	.035	− .497***
SEP ← Spiritual Formation	− .001	.011	− .013
SEN ← Spiritual Formation	.043	.030	.147
SEP ← Spiritual Being	− .003	.011	− .034
SEN ← Spiritual Being	− .024	.029	− .087
SEP ← Faith	.005	.011	.066
SEN ← Faith	.069	.028	.308*
SEP ← Online Being	− .006	.007	− .062
SEN ← Online Being	− .038	.018	− .133*
SEP ← Community	.041	.014	.302**
SEN ← Community	.040	.028	.106
SEP ← Material Values	.035	.013	.249**
SEN ← Material Values	− .015	.027	− .037
SEP ← Sense of Life	− .011	.009	− .116
SEN ← Sense of Life	− .082	.024	− .320***
SEP ← Happiness	.033	.011	.253**
SEN ← Happiness	− .063	.024	− .171**
SEP ← Dialog with Self	.008	.009	.083
SEN ← Dialog with Self	− .010	.024	− .035
SEP ← Spiritual Deed	− .012	.012	− .118
SEN ← Spiritual Deed	− .071	.031	− .243*
Measurement level (factors)			
SelfEs10 ← SEP	1.000		.304***
SelfEs4 ← SEP	2.026	.471	.729***
SelfEs3 ← SEP	2.219	.512	.789***
SelfEs2 ← SEP	1.258	.314	.517***
SelfEs1 ← SEP	1.354	.338	.517***
SelfEs9 ← SEN	1.000		.684***
SelfEs8 ← SEN	− 1.095	.119	.709***
SelfEs7 ← SEN	.836	.098	.644***
SelfEs6 ← SEN	1.000	.112	.672***
SelfEs5 ← SEN	.851	.107	.583***

Statistical significance *p < .05; **p < .01; ***p < .001

### Model Testing—Structural Equation Modelling (SEM)

The fit of the proposed structural model was satisfactory (*χ*^2^ = 276.99, df = 131, *p* > .05; RMSEA = .06; 90% CI .05/.08; CFI = .94; IFI = .94) and explained 41% of the variance in the self-esteem negative and 32% of variance in self-esteem positive.

Table 4 presents standardised and unstandardised regression weights for the specified structural equation *Model of Association of Self*-*Esteem with Spiritual Values* (Fig. 1). As can be observed, self-esteem positive was significantly predicted by Community (*β* = .30, *p* < .01), Material Values (*β *= .25, *p* < .01), and Happiness (*β *= .25, *p* < .01). In relation to self-esteem negative, the significant predictors estimated in SEM were prayer (*β *= − .50, *p* < .001), faith (*β *= .31, *p* < .05), body (*β *= − .13, *p* < .05), ultimate truth (*β *= − .32, *p* < .001), Happiness (*β *= − .17, *p* < .01), and deed (*β *= − .24, *p* < .05).

## Findings, Discussion, and Conclusion

### Findings

The aim of this study was twofold: Firstly, it was to investigate the construct validity and dimensionality of the Rosenberg self-esteem scale (RSES) (Rosenberg 1965) and its association with spiritual values, as a twofold structure divided on self-esteem positive (SEP) and self-esteem negative (SEN). The findings confirmed that SE functions as a twofold construct rather than as a monolithic form. This is in line with previous research of Debowska et al. (2017), Hyland et al. (2014), McKay et al. (2014), and Boduszek et al. (2012, 2013). Secondly, it was to investigate a correlation between SE and spiritual factors. Although there are extensive studies examining the association of religiosity–spirituality with self-esteem, this research is the first attempt to explore the theme using SE as a twofold structure (SEP and SEN). *The Model of Association of Self*-*Esteem with Spiritual Values* (ASESV) indicates that association of spiritual values with SEP is a reverse of the scores associated with SEN and there is no indication that SEP and SEN are bipolar constructs, as the scores are in line with the direction of unitary SE construct.

This study has demonstrated that eight, out of twelve, independent variables are deemed to be significant in predicting SE. Four, out of twelve, revealed no significant association with SE: They are God, Spiritual Formation, Spiritual Being, and Dialogue with Self. Some relationships with SEP were not significant when compared with SEN. The findings of this study confirm that there are a number of spiritual areas which influence people’s self-esteem. Among them, the strongest predictor is Happiness, associating with both SEP and SEN, followed by; Spiritual Deed, Sense of Life, Online Being, Faith, and Prayer, that predict only SEN. Material Values and Community predict only SEP. The study also finds that there are several religious–spiritual predictors that do not significantly predict SE.

## Discussion

The results of this research have produced ambiguous findings. On the one hand, it could be concluded that religious and spiritual engagement has a positive influence on self-evaluation. Yet, on the other hand, the evidence is inconclusive. When looking at the significant predictors of SE, it can be said that there is a positive correlation between SE and spirituality–religiosity. This is in line with research undertaken by Chamberlain and Zika (1992), and Levin and Tobin (1995), who found that religiosity has a positive influence on a person’s mental health and well-being. The study of Diener et al. (1999) could also be comparable as it demonstrates the importance of Happiness in self-evaluation, as Happiness is the strongest predictor in ASESV. Results obtained by Poloma and Pendleton (1990) found that some religious scales of religious satisfaction, frequency of prayer, prayer experience, relationship with God are important for well-being. Current research partially confirmed previous studies of Benson and Spilka (1973), which found positive association of self-esteem with religious values. They differ, however, on the association of God’s image with self-esteem. This is because the ASESV does not find God as a significant predictor. However, it should be noted that the study of Benson and Spilka (1973) used a much less diversified sample of one-hundred and twenty-eight catholic participants.

Johnson (2001), Huls and Waaijman (2005) suggested that participation in Community is important for SE. The ASESV model confirmed that, but it has specified its positive correlation only with SEP. Zaleski (2002) and Hayles (1993, 2002) suggested that virtual reality largely implemented by technological progress influences people’s spiritual life. Our results show that people who only respond positively to SEN participate less in online social activities. Maas (2005) suggested that the lack of reference regarding the search for the sense of life correlates with self-esteem. This is found again and confirmed through this research. This research concretises its correlation with SEN, but not with SEP. Lower SE generates a lower craving in search for meaning in life, or put another way, a lower desire for finding meaning in life deteriorates SE. The specificity of this relationship would need to be further investigated, perhaps in a longitudinal study. The research of Emmons et al. (1998) examined related aspects and proposed a very developed and complex study, finding that formation is important in self-evaluation. Such evidence was not confirmed by the current findings.

The observation of nonsignificant predictors in ASESV provides inconclusive yet intriguing evidence of to the hitherto inferences. This gives rise to the question: Why such important religious–spiritual variables such as God, Spiritual Formation, Spiritual Being, and Dialogue with Self have no significant correlation with SE? And why is it that it is only Happiness which correlates significantly with both SEP and SEN? Previously, Bahr and Martin (1983) found little relationship between self-esteem and religiosity, and their study produced inconsistent findings. The study of Krause (1995) also found that it is difficult to establish a clear association of self-esteem with religiosity. Nonetheless, Krause found that people with little religious commitment reveal lower self-worth from those who engage more.

A limitation of this research is that the sample was drawn only from the population of the Republic of Ireland which remains predominately Roman Catholic. A sample dominated by other belief groups or more equalised population could present different responses. Therefore, the inclusion of populations from different countries and potentially a larger number of participants could produce an interesting contribution to this study.

## Conclusion

The relevance of this research has at least three dimensions. Firstly, this study confirmed previous findings that spirituality–religiosity have positive influence on peoples’ self-evaluation. Secondly, clear and linear correlation between spirituality and SE cannot be produced. The reason for this is that only some aspects of spirituality significantly correlate with SE, whilst others, appearing as vital for spirituality, for instance, God, Spiritual Being, Spiritual Formation, Dialogue with Self, do not. Generally, it seems problematic to produce an unequivocal statement other than to say that spirituality has a positive association with SE, whilst components of spirituality and religiosity influence it individually and fragmentally. Thirdly, the originality and innovation of this research are the application of twofold construct of SE with SEP and SEN rather than the hitherto investigations of the unitary model within the context of spiritual and religious values. This discloses interesting specifications of correlations with both or specifically each one of the components of RSES, which is SEP or SEN, instead of generalised results. This non-simplified scrutiny opens the way towards some insightful conclusion that not only do people react oppositely but also differently to SEP and SEN. Both constructs represent a structure that is statistically internally consistent as a whole, which means it represents a very close paradigm. However, the findings clearly indicate that SEP and SEN are not only oppositely but also diversely perceived by respondents most of the time without same–opposite reaction to the contrary construct of RSES, the Happiness the only exception in this study. Therefore, one of the vital findings of this research is that SEP or SEN may be applied independently as a singular variable in the research, and that the application of RSES as a one, ten-question scale construct may appear statistically fitting, whilst at the same time be considered, by some as inappropriate and confusing for the research findings.
